# Which Psycho‐Oncological Interventions Are Applied in an Acute Care Hospital in Germany?—An Exploratory Retrospective Cross‐Sectional Single Center Study

**DOI:** 10.1002/pon.70284

**Published:** 2025-10-01

**Authors:** Marietta Lieb, Martina Madl, Eva Morawa, Yesim Erim

**Affiliations:** ^1^ Department of Psychosomatic Medicine and Psychotherapy Friedrich‐Alexander University Erlangen‐Nürnberg (FAU) Erlangen Germany

**Keywords:** cancer, interventions, oncology, psycho‐oncological service

## Abstract

**Background:**

A psycho‐oncological service (POS) is mandatory for certified cancer centers in Germany. However, research on what specific interventions are used in acute cancer care is lacking.

**Aims:**

The aim of our study was to quantify the use and context‐specific application patterns of psycho‐oncological interventions used during psycho‐oncological contacts.

**Methods:**

This is an exploratory retrospective cross‐sectional single‐center study, analyzing data from the POS from the University Hospital in Erlangen from the year 2023. In the hospital's digital documentation system, each psycho‐oncological session is recorded and the applied interventions are specified according to a self‐developed taxonomy. We analyzed the number and type of applied interventions and examined their association with gender, age, cancer entity and tumor situation.

**Results:**

In 2023, 2636 patients had 5615 psycho‐oncological contacts. Interventions most frequently applied in sessions lasting more than 10 min were *Exploration of the current state, Establishing the relationship, Validating, Normalizing* and *Working on resources*. *Normalization* was more often used for female than male patients. *Psycho‐education*, *Establishing the relationship, Exploration of current state*, *Counseling* and *Normalizing* were more frequently applied for younger patients. *Existential methods* were used predominantly for patients with metastasized initial disease. Patients with hematological cancer had the highest rates for most interventions.

**Conclusions:**

There are interventions that are inherent to nearly all psycho‐oncological contacts, forming the core components of psycho‐oncological contacts. Interventions less frequently used seem to be applied according to the patients' needs. Psycho‐oncological support therefore means flexible utilization of therapeutic interventions, making it a relevant part of comprehensive oncological treatment.

## 1 Background

A cancer diagnosis and its treatment can be a drastic break in a cancer patient's life, impacting both physical and psychological well‐being. This often results in heightened levels of emotional distress [1], while approximately one third of all patients fulfills the criteria for a mental disorder [2]. The most frequent mental disorders in cancer patients are affective disorders, anxiety disorders and adjustment disorders [3]. The prevalence of these mental health issues is significantly higher among cancer patients than in the general population [2], therefore leading to an increased need for psychological support in this group. A total of 28.9% of cancer patients reported that they have used psychotherapy or psychological counseling due to cancer‐related distress [4].

In comprehensive (certified) cancer centers in Germany (CCCs), a psycho‐oncological service (POS) is mandatory in order to ensure high quality professional care for all cancer patients [5]. The POS can be established either as a consultation (on demand) or liaison service (psycho‐oncologists are constantly on site) [5], providing low‐threshold access to psycho‐oncological support at any point of treatment for every patient. According to the psycho‐oncological guideline [3], all patients should be screened and informed about the POS upon hospital admission and their possibility to use it if necessary. As the POS is included as a regular service in the German health care system, it is automatically covered for patients with statutory health insurance and thus free of cost. In Germany, the psycho‐oncological qualification requires a university degree in psychology or medicine and an additional certified training for psycho‐oncology. Therefore, the employees of the POS are able to provide high standard patient care comprising professional psychological cancer‐related knowledge and experience.

The psycho‐oncological approach is mostly of supportive nature and covers a wide spectrum of tasks that aim to assist cancer patients and their relatives in dealing with psychosocial consequences of cancer, to reduce mental distress, to strengthen personal resources and to maintain the highest possible level of autonomy and quality of life [3, 6, 7]. In case of subsyndromal distress, patients profit most from psychoeducation, relaxation techniques, art therapy, psychosocial counseling and psychotherapeutic methods, while in case of diagnosed mental disorders, treatment according to the respective guidelines is indicated [3]. Psycho‐oncology uses many methods draws on various psychotherapeutic approaches, such as cognitive behavioral therapy, psychodynamic therapy, person‐centered psychotherapy, systemic psychotherapy, interpersonal therapy etc. [7], therefore psycho‐oncological interventions applied in routine care differ immensely. The effectiveness of psycho‐oncological support has been proven in several studies by now [8, 9, 10]; however, due to the broad range of psycho‐oncological interventions, comparisons between studies are challenging.

Despite these findings, little is known about the specific interventions used in acute cancer care. Therefore, similar to Michie et al.’s taxonomy of behavior change techniques [11], we have developed a comprehensive and structured taxonomy of psycho‐oncological interventions in an acute care hospital in Germany in order to obtain a standardization for research and applied care [12]. The taxonomy included 22 psycho‐oncological intervention techniques that were classified into five superordinate categories (1. Contact/information; 2. Anamnesis/assessment of state of health; 3. Psychotherapeutic interventions; 4. Psychotherapeutic interventions specific to the psycho‐oncological setting and 5. Psychotherapeutic behavior/relationship).

With this follow‐up exploratory study, we aimed to quantify the use and context‐specific application patterns of psycho‐oncological interventions used during psycho‐oncological contacts. Our research questions were as follows:

1. Which interventions are applied and how often?
2. Do applied interventions differ according to gender, age, diagnosis and tumor situation?

## 2 Methods

### 2.1 Setting and Procedure

The POS of the University Hospital in Erlangen is part of the Department of Psychosomatic Medicine and Psychotherapy. For 16 oncological centers, the hospital has established liaison services, while there are consultation services for several other centers on demand. Every psycho‐oncological contact is recorded in the hospital's internal digital documentation system of the POS, using the self‐developed taxonomy list and indicating which interventions are applied for the respective sessions.

### 2.2 Data Collection

For analysis, we used the most current data from the year 2023. The development process of the taxonomy, the list of interventions as well as the definitions of the respective interventions can be found in Madl et al. [12].

Because the conversations were documented as part of standard care, patients were not explicitly asked to participate in the study.

### 2.3 Ethics

In the course of the development of the taxonomy [12], institutional ethics committee approval was obtained from the Clinical Ethics Committee of the University Hospital of Erlangen.

No informed consent of the patients for our study was required as our study analyzed anonymously standard care data from the university hospital, patients were not explicitly asked to participate in the study.

### 2.4 Statistical Analysis

All statistical analyses were performed with IBM SPSS statistics, version 28. For descriptive statistics, we displayed means, standard deviations and ranges. For comparisons of gender, we used independent *t*‐Tests or Welch tests depending on the fulfillment of statistical requirements. Effect sizes were interpreted via Cohen's *d* (*d* ≈ 0.2 = small effect, *d* ≈ 0.5 = medium effect, *d* ≈ 0.8 = large effect [13]). For comparisons of tumor situation and diagnosis, we performed ANOVAs/Welch‐Tests and proceeded with post hoc tests (Tukey or Games‐Howell). In case of severe deviation from heteroscedasticity, we used the Bootstrapping method with 1000 iterations. In case of very unevenly sized groups (e.g., tumor situation), we used the Brown‐Forsythe Test instead of the Welch‐test. For the interpretation of effect size, we used Eta^2^ (*η* ^2^ ≈ 0.01 = small effect, *η* ^2^ ≈ 0.06 = medium effect, *η* ^2^ ≈ 0.14 = large effect [13]). Correlations were calculated with the Pearson correlation coefficient. For all statistical analyses, a significance level of *p* < 0.05 was predefined.

## 3 Results

### 3.1 Frequency and Duration of Psycho‐Oncological Contacts

In the year 2023, 2636 patients had 5615 psycho‐oncological contacts. The average contact frequency per patient was *M* = 2.13 (SD = 2.39) with a range of 1–43. The average duration per contact was *M* = 24.82 (SD = 19.43) minutes, with a range of 1–180, while the average duration per patient was *M* = 50.73 (SD = 96.03, range 1–2125). A total of 1934 (34.4%) of all contacts were short sessions of 0–10 min, 1884 (33.6%) were sessions of 11–30 min and 1395 (24.8%) were sessions of 30 min and longer. For 402 (7.2%) contacts, no time was indicated.

Female patients had significantly more contacts than male patients (Female: *M* = 2.23, SD = 2.52, Male: *M* = 1.98, SD = 2.18, *t*(2634) = −2.58, *p* = 0.008, Cohen's *d* = −0.103), while the contact duration did not differ (Female: *M* = 20.99, SD = 13.79, Male: *M* = 20.25, SD = 13.73, *t*(2549) = −1.32, *p* = 0.186, Cohen's *d* = −0.054). Younger patients had significantly more (*r* = −0.143, *p* < 0.001) and longer contacts (*r* = −0.164, *p* < 0.001) than older patients.

### 3.2 Initiative and Type of Psycho‐Oncological Contacts

Of all contacts, 2522 (44.9%) were initiated by the patients themselves, 1345 (24.0%) by the psycho‐oncologists, 726 (12.9%) by physicians, 359 (6.4%) by nurses, 200 (3.6%) by relatives of the patients, 26 (0.5%) by other persons such as patient guides, and 11 (0.2%) by the social service. For the remaining 426 (7.6%) cases, the initiative was unknown.

Most of the psycho‐oncological contacts (3376, 60.1%) were individual face‐to‐face sessions with the patient. Another 1009 (18.0%) contacts were phone calls with patients. 498 (8.9%) contacts were care for relatives (face‐to‐face, phone calls and E‐Mails) and 209 (3.7%) were face‐to‐face couple sessions. The remaining 521 (9.3%) contacts were either E‐Mail contacts with the patient or were not specified.

### 3.3 Sociodemographic and Medical Data of Patients

The sociodemographic data and diagnoses of all patients receiving psycho‐oncological care in 2023 is displayed in Table 1.

**TABLE 1:** Sociodemographic and medical data (*N* = 2636).

		*N* = 2636
Gender, *n* (%)	Female	1585 (60.1%)
	Male	1051 (39.9%)
Age, in years, *M* (SD), range		62.35 (15.02), 18.02–100.01
Relationship, *n* (%)	Yes	1366 (51.8%)
	No	368 (14.0%)
	Unspecified/unknown^a^	902 (34.2%)
Children, *n* (%)	Yes	1252 (47.5%)
	No	289 (11.0%)
	Unspecified/unknown	1095 (41.6%)
Employment status, *n* (%)	Unemployed	21 (0.8%)
	Employed	748 (28.4%)
	Housework	23 (0.9%)
	Retired	716 (27.2%)
	Apprentice/student	28 (1.1%)
	Unspecified/unknown	959 (36.4%)
Cancer entity, *n* (%)	Colon/Rectum	171 (6.5%)
	Brain	218 (8.3%)
	Gynecological	235 (8.9%)
	Hematological	188 (7.1%)
	Skin	299 (11.3%)
	Head/neck	251 (9.5%)
	Lung/bronchial	99 (3.8%)
	Visceral^b^	154 (5.8%)
	Breast	533 (20.2%)
	Prostate/testicular	93 (3.5%)
	Urological	46 (1.7%)
	Soft tissue sarcoma	70 (2.7%)
	Other	91 (3.5%)
	No tumor diagnosis^c^	85 (3.2%)
	Unspecified/unknown	103 (3.9%)
Tumor situation, *n* (%)	Initial disease (non‐metastasized)	1652 (62.7%)
	Initial disease (metastasized)	227 (8.6%)
	Metastasized	173 (6.6%)
	Relapse	124 (4.7%)
	Remission^d^	2 (0.1%)
	Secondary tumor	82 (3.1%)
	Chronic^d^	20 (0.8%)
	Follow‐up care	89 (3.4%)
	Unspecified/unknown	267 (10.1%)

^a^ Especially in short contacts, usually not all sociodemographic information is assessed.

^b^ Includes stomach, small intestine, esophagus, pancreas, liver, gall bladder, bile duct.

^c^ Patients only have a suspected diagnosis and/or it is unclear as to whether the tumor is benign or malignant.

^d^ Excluded for further analyses due to small sample size.

### 3.4 Frequency of Applied Interventions

The frequency of applied interventions is presented in Table 2.

**TABLE 2:** Frequency of applied interventions (*N* = 32,823).

Cluster	Intervention	Total frequency	Percentage frequency of all interventions (%)	Percentage frequency of all contacts (%)
		*N* = 32,823		
1. Contact/information	Introduction	3105	9.5%	55.3%
	Counseling	788	2.4%	14.0%
2. Anamnesis/assessment of state of health	Exploration of current state	4187	12.8%	74.6%
	Survey of disease progression/medical history	2356	7.2%	42.0%
	Defining the therapeutic mandate	3635	11.1%	64.7%
	Assessment of suicidality	87	0.3%	1.5%
3. Psychotherapeutic interventions	Psycho‐education	825	2.5%	14.7%
	Working on emotions	420	1.3%	7.5%
	Using cognitive methods	840	2.6%	15.0%
	Relaxation techniques/guided imagery	144	0.4%	2.6%
	Working on resources	2410	7.3%	42.9%
	Development of social skills	286	0.9%	5.1%
4. Psychotherapeutic interventions specific to the psycho‐oncological setting	Accompaniment	141	0.4%	2.5%
	Crisis intervention	31	0.1%	0.6%
	Life review	122	0.4%	2.2%
	Bearing of emotions (holding/containing)	1497	4.6%	26.7%
	Coping with fear of progression/recurrence	333	1.0%	5.9%
	Existential methods	131	0.4%	2.3%
	Grief work	289	0.9%	5.1%
5. Psychotherapeutic behavior/relationship	Establishing the relationship	4107	12.5%	73.1%
	Normalizing	3213	9.8%	57.2%
	Validating	3876	11.8%	69.0%

We found a significant correlation between the total number of interventions applied per contact and the duration of the contact (*r* = 0.613, *p* < 0.001). Longer contacts were associated with more interventions. We further found that some interventions were significantly more often applied during shorter contacts (0–10 min), such as *Introduction* (F(2,3252.42) = 168.88, *p* < 0.001, *η* ^2^ = 0.058) and *Defining the therapeutic mandate* (F(2,3050.43) = 311.49, *p* < 0.001, *η* ^2^ = 0.091). All other interventions were significantly more often applied in longer sessions (lasting more than 10 min, *p* < 0.001, results not shown).

We also found significant positive correlations between the number of contacts and the number of each applied intervention (e.g., *Life review: r* = 0.226, *p* < 0.001, *Exploration of current state*: *r* = 0.926, *p* < 0.001).

### 3.5 Differences in Applied Interventions According to Gender, Age, Cancer Entity and Tumor Situation

Differences of applied interventions according to gender as well as correlations of the frequency of interventions with age are displayed in Table S1.

In Table 3, all ANOVAs that were computed regarding the differences in applied interventions according to diagnosis are displayed. We found significant differences in diagnoses for all applied interventions, except for *Crisis intervention*. For all significant ANOVAs, we proceeded with follow‐up post‐hoc tests for the interventions that were used most and least.

**TABLE 3:** ANOVAs with post hoc tests for differences in applied interventions according to diagnosis.

Intervention	ANOVA	Diagnose with highest rate	Significant post hoc tests compared to highest rate^a^	Diagnose with lowest rate	Significant post hoc tests compared to lowest rate^a^
		M (SD)		M (SD)	
Introduction	F(1,613) = 5.59, *p* < 0.001, *η* ^2^ = 0.030	Soft tissue sarcoma	Hematological	Hematological	All, except for skin, prostate, urological
		1.50 (1.13)		0.88 (0.72)	
Counseling	F(13,594) = 17.79, *p* < 0.001, *η* ^2^ = 0.144	Hematological	All, except for soft tissue sarcoma	Head/neck	Brain, hematological, skin, lung, breast, others, no diagnosis
		1.41 (1.84)		0.03 (0.22)	
Exploration of current state	F(13,613) = 5.55, *p* < 0.001, *η* ^2^ = 0.026	Hematological	All, except for gynecologic, visceral, head/neck, others	Prostate	Colon/rectum, gynecological, hematological, skin, visceral, breast, head/neck, no diagnosis
		2.52 (2.79)		0.87 (1.17)	
Survey of disease progression	F(13,612) = 8.67, *p* < 0.001, *η* ^2^ = 0.044	Visceral	Brain, gynecological, breast, prostate	Breast	Colon/rectum, hematological, skin, visceral, head/neck
		1.43 (1.75)		0.58 (1.09)	
Defining the therapeutic mandate	F(13,636) = 10.51, *p* < 0.001, *η* ^2^ = 0.026	Gynecological	Brain, prostate, urological	Urological	Colon/rectum, gynecological, skin, no diagnosis, visceral, breast, soft tissue sarcoma, head/neck
		1.66 (2.37)		0.85 (0.56)	
Assessment of suicidality	F(13,603) = 1.78, *p* = 0.044, *η* ^2^ = 0.009	Lung	^b^	Gynecological	^b^
		0.11 (0.34)		0.008 (0.09)	
Psychoeducation	F(13,611) = 8.52, *p* < 0.001, *η* ^2^ = 0.053	Hematological	All, except for soft tissue sarcoma	Gynecological	Brain, hematological
		0.93 (1.14)		0.14 (0.43)	
Working on emotions	F(13,595) = 8.57, *p* < 0.001, *η* ^2^ = 0.063	Hematological	All, except for no diagnosis, visceral, prostate, others, soft tissue sarcoma	Head/neck	Hematological, prostate
		0.66 (1.35)		0.028 (0.19)	
Using cognitive methods	F(13,601,64) = 14.25, *p* < 0.001, *η* ^2^ = 0.075	Hematological	All, except for soft tissue sarcoma	Head/neck	Colon/rectum, gynecological, hematological, skin, visceral, breast
		1.26 (2.02)		0.05 (0.26)	
Relaxation techniques/guided imagery	F(13,609) = 2.12, *p* = 0.012, *η* ^2^ = 0.043	Hematological	All, except for no diagnosis, soft tissue sarcoma	Urological	Hematological
		0.29 (0.79)		0.02 (0.15)	
Working on resources	F(13,614) = 6.84, *p* < 0.001, *η* ^2^ = 0.035	Hematological	Brain, skin, breast, prostate, urological, head/neck	Head/neck	Colon/rectum, hematological, visceral
		1.52 (1.94)		0.56 (1.38)	
Development of social skills	F(13,610) = 3.81, *p* < 0.001, *η* ^2^ = 0.019	Hematological	Brain, gynecological, breast	Gynecological	Hematological, head/neck
		0.27 (0.78)		0.03 (0.24)	
Accompaniment	F(13,2519) = 4.62, *p* < 0.001, *η* ^2^ = 0.023	Brain	Skin, prostate, head/neck	Skin	Brain, breast
		0.19 (0.68)		0.00 (0.00)	
Crisis intervention	F(13,2519) = 0.970, *p* = 0.479, *η* ^2^ = 0.005	—	^b^	—	^b^
Life review	F(13,2519) = 4.36, *p* < 0.001, *η* ^2^ = 0.022	No diagnosis	Brain, gynecological, breast, urological, head/neck	Urological	All except for gynecological, prostate, soft tissue sarcoma
		0.15 (0.52)		0.00 (0.00)	
Bearing of emotions (holding/containing)	F(13,618) = 11.09, *p* < 0.001, *η* ^2^ = 0.056	Soft tissue sarcoma	^b^	Urological	Colon/rectum, hematological, skin, no diagnosis, visceral, others
		1.32 (3.01)		0.26 (0.49)	
Coping with fear of progression/recurrence	F(13,635) = 4.22, *p* < 0.001, *η* ^2^ = 0.023	Skin	All, except for hematological, visceral, others, soft tissue sarcoma	Urological	Skin
		0.34 (0.99)		0.02 (0.15)	
Existential methods	F(13,2519) = 3.69, *p* < 0.001, *η* ^2^ = 0.019	Soft tissue sarcoma	^b^	Gynecological	Colon/rectum, hematological, skin, lung, visceral, breast, prostate
		0.20 (1.56)		0.00 (0.00)	
Grief work	F(13,600) = 3.53, *p* < 0.001, *η* ^2^ = 0.026	Soft tissue sarcoma	^b^	Skin	Hematological
		0.44 (1.94)		0.05 (0.32)	
Establishing the relationship	F(13,615) = 5.25, *p* < 0.001, *η* ^2^ = 0.025	Hematological	All, except for gynecological, visceral, others, soft tissue sarcoma, head/neck	Prostate	Colon/rectum, gynecological, hematological, skin, visceral, breast, head/neck
		2.45 (2.78)		0.87 (1.20)	
Normalizing	F(13,614) = 13.85, *p* < 0.001, *η* ^2^ = 0.042	Hematological	Brain, skin, no diagnosis, prostate, urological	Brain	All, except for prostate, urological, soft tissue sarcoma
		1.89 (2.28)		0.52 (1.02)	
Validating	F(13,613) = 7.93, *p* < 0.001, *η* ^2^ = 0.034	Hematological	All except for gynecological, visceral, others, soft tissue sarcoma, head/neck	Prostate	Gynecological, hematological, skin, visceral, breast, head/neck
		2.46 (2.89)		0.84 (1.21)	

^a^ Significant difference on a significance level of *p* < 0.05 for the listed diagnoses.

^b^ No significant differences in the post hoc tests.

Especially patients with hematological cancer had the highest rates of most applied interventions such as *Counseling* (large effect), *Working on emotions* (medium effect), *Using cognitive methods* (medium effect) and *Psychoeducation* (small to medium effect). A small effect was observed for the interventions *Exploration of the current state, Relaxation techniques, Working on resources, Development of social skills, Establishing the relationship, Normalizing* and *Validating.* Patients with urological cancer had the lowest rates of *Bearing of emotions* (small to medium effect), *Relaxation techniques* (small effect), and *Coping with fear of progression* (small effect), and they had no *Life review* at all (small effect). Patients with gynecological cancer had the lowest rates for *Psychoeducation* (small to medium effect), *Development of social skills* (small effect), and no *Existential methods* at all (small effect).

We further computed several ANOVAs in order to analyze differences of applied interventions depending on the tumor situation. We found no significant difference for the tumor situation for the following interventions: *Counseling* (F(5,440) = 1.04, *p* = 0.394), *Exploration of current state* (F(5,744) = 1.89, *p* = 0.095), *Defining the therapeutic mandate* (F(5,722) = 1.63, *p* = 0.150), *Assessment of suicidality* (F(5,635) = 2.23, *p* = 0.050), *Psychoeducation* (F(5,732) = 2.02, *p* = 0.074), *Using cognitive methods* (F(5,588) = 1.55, *p* = 0.172), *Relaxation techniques* (F(5,267) = 1.35, *p* = 0.244), *Accompaniment* (F(5,762) = 0.58, *p* = 0.715), *Crisis intervention* (F(5,2341) = 0.369, *p* = 0.870), *Life review* (F(5,579) = 1.98, *p* = 0.08), *Establishing the relationship* (F(5,734) = 1.34, *p* = 0.246), *Normalizing* (F(5,710) = 1.39, *p* = 0.224) and *Validating* (F(5,724) = 1.58, *p* = 0.165). For *Survey of disease progression* (F(5,764) = 2.76, *p* = 0.017, *η* ^2^ = 0.008)*, Development of social skills* (F(5.528) = 2.26, *p* = 0.047, *η* ^2^ = 0.006) and *Coping with fear of progression* (F(5,519) = 2.99, *p* = 0.011, *η* ^2^ = 0.008), the ANOVAs were significant, while follow‐up post hoc tests did not reveal any significant differences between tumor situations.

However, for *Introduction*, *Working on emotions*, *Working on resources*, *Bearing of emotions*, *Existential methods* and *Grief work*, ANOVAS were significant with significant post hoc tests. The results are displayed in Table 4. For Initial disease (metastasized), the depicted interventions were most frequent (except for *Bearing of emotions*), while they were lowest for follow‐up care (except for *Working on emotions*). However, only for *Existential methods* a small effect was detected, all other significant differences showed a marginal effect.

**TABLE 4:** Significant ANOVAs with post hoc tests for differences in applied interventions according to tumor situation.

Intervention	ANOVA	Tumor situation with highest rate	Post hoc tests compared to highest rate*	Tumor situation with lowest rate	Post hoc tests compared to lowest rate*
		M (SD)		M (SD)	
Introduction	F(5,753) = 3.83, *p* = 0.002, *η* ^2^ = 0.007	Initial disease (metastasized)	Follow‐up care	Follow‐up care	Initial disease (metastasized),
		1.37 (0.95)		1.01 (0.61)	Initial disease (non‐metastasized)
Working on emotions	F(5,570) = 4.74, *p* < 0.001, *η* ^2^ = 0.006	Initial disease (metastasized)	Initial disease (non‐metastasized), secondary tumor	Secondary tumor	Initial disease (metastasized)
		0.39 (1.08)		0.05 (0.35)	
Working on resources	F(5,756) = 4.41, *p* < 0.001, *η* ^2^ = 0.009	Initial disease (metastasized)	Initial disease (non‐metastasized), follow‐up care	Follow‐up care	Initial disease (metastasized)
		1.35 (2.09)		0.66 (1.29)	
Bearing of emotions (holding/containing)	F(5,730) = 2.97, *p* = 0.011, *η* ^2^ = 0.006	Metastasized	^a^	Follow‐up care	Initial disease (metastasized)
		0.80 (1.64)		0.42 (0.74)	
Existential methods	F(5,234) = 7.59, *p* < 0.001, *η* ^2^ = 0.011	Initial disease (metastasized)	Follow‐up care	Follow‐up care	Initial disease (non‐metastasized), initial disease (metastasized), metastasized, relapse
		0.18 (0.58)	Initial disease (non‐metastasized), secondary tumor, relapse	0.00 (0.00)	
Grief work	F(5.562) = 3.07, *p* = 0.01, *η* ^2^ = 0.009	Initial disease (metastasized)	Follow‐up care	Follow‐up care	Initial disease (metastasized)
		0.24 (0.84)		0.06 (0.28)	

^a^ No significant differences in the post hoc tests.

## 4 Discussion

In this study, we quantified the use and context‐specific application patterns of psycho‐oncological interventions applied during psycho‐oncological contacts on the basis of our previously published taxonomy of psycho‐oncological interventions in an acute care hospital [12]. After the descriptive display of the frequency and duration of psycho‐oncological contacts as well as the treated patients, we comprehensively depicted the applied interventions and examined their association with gender, age, cancer entity and tumor situation.

In the course of one year, 2626 patients had made use of 5615 sessions of the psycho‐oncological service at the University Hospital in Erlangen, whereby the majority of patients was female (60.1%). Female and younger patients had more psycho‐oncological contacts than male and older patients, which is well in line with other studies [4, 14, 15]. The increased proportion of breast cancer patients (20.25%) in our study is also comparable to previous research [4, 15], providing a representative sample for our study.

The most frequently applied psycho‐oncological interventions were *Exploration of the current state*, which was used in 74.6% of all psycho‐oncological contacts, followed by *Establishing the relationship*, applied in 73.1% of all contacts, *Validating*, administered in 69.0% of all contacts and *Normalizing*, used in 57.2% of contacts. *Establishing the relationship* implies the display of an empathic attitude, *Exploration of the current state* involves active listening and targeted questioning [12], while *Validation* and *Normalization* comprise appreciation for the patient's feelings and thoughts. These interventions are core components of psychotherapy and psycho‐oncological support. *Establishing the relationship*, *Exploration of the current state* and *Validation* are structuring elements of psycho‐oncological care and therefore providing the frame for subsequent interventions. Especially the relationship has long been regarded as the most effective factor for therapy [16, 17].

Other interventions that were frequently applied were *Introduction, Survey of the disease progression, Defining the therapeutic mandate* and *Working on resources. Introduction* and *Defining the therapeutic mandate* were significantly more often applied in short contacts (less than 10 min), in order to evaluate the psycho‐oncological need of the patient. Since not all contacted patients do need psycho‐oncological support or the need for support indicated at screening has already decreased, contacts can be kept time efficient or patients can be referred to other service (e.g., social service etc.). *Working on resources* was applied in nearly every second contact (42.9%), which also seems to be an inherent component for psycho‐oncological work. It includes reinforcement of positive actions, development of coping strategies and activating social support [12]. Since cancer has a dramatic impact on people's lives, the situation often exceeds available emotional and social resources and patients often do not have access to adequate coping strategies. The exploration and development of resources is thus an essential part of psycho‐oncological sessions. A variety of resources and adaptive coping strategies have repeatedly been associated with an increased quality of life, better mental and general health in cancer patients [18, 19, 20].

All other interventions were applied less frequently and therefore most likely flexibly adopted according to the situation and the patient's needs and characteristics. For instance, some interventions differed according to gender, age, cancer entity and tumor situation. For gender, we found significant differences for *Exploration of current state, Defining the therapeutic mandate, Relaxation techniques, Working on resources, Coping with fear of progression, Establishing the relationship, Normalizing* and *Validating*. All were significantly more often used in women. However, all effect sizes were minor (*d* < 0.15). Solely for *Normalizing* the effect size could be interpreted as small, when rounded (*d* = 0.19). *Normalizing* includes the reinsurance that thoughts and emotions are situationally adequate. Previous research suggests different coping strategies with cancer in women and men [21], which may also lead to differences in applied interventions. Regarding age, we further found several significant associations, also with minor (*r* < 0.10) or small effects (*r* > 0.10), though. All interventions from the *Psychotherapeutic behavior* cluster were significantly more often used for younger patients (*Establishing the relationship*: *r* = −0.131, *Normalizing*: *r* = −0.118, *Validating: r* = −0.113). Moreover, *Counseling* (*r* = −0.123), *Exploration of current state* (*r* = −0.128), *Psycho‐education* (*r* = −0.148), *Using cognitive methods* (*r* = −0.103), *Relaxation techniques* (*r* = −0.108) and *Working on resources* (*r* = −0.115) were more frequently applied for younger patients.

Regarding cancer entity, we found significant differences for all applied interventions with small (*η* ^2^ = 0.009) to large (*η* ^2^ = 0.144) effect sizes, except for *Crisis intervention.* Patients with hematological cancer had the highest rate of most interventions, *Counseling* showing a large effect, *Working on emotions* and *Using cognitive methods* a medium effect. One explanation could be that patients with this diagnosis also have the highest frequency of contacts due to high distress during isolation [14] and thus more interventions. However, not all interventions were most frequent in this diagnosis, for example patient with brain tumors most often had *Accompaniment* and patients with visceral cancer *Survey of disease progression*. Patients with brain tumors are often most cognitively impaired, therefore a close personal support seems most relevant in this patient group. Visceral cancer, especially pancreatic cancer often have a fast and fatal course, which could be the reason why *Survey of disease progression* is used most often. For patients with gynecological and urological cancer, many interventions were applied least frequently. Patients with urological cancer had the lowest rates especially for *Bearing of emotions* (small to medium effect). Patients with gynecological cancer had the lowest rates in particular for *Psychoeducation* (small to medium effect).

There were only few interventions that significantly differed according to tumor situation, and even less that had an interpretable effect size. *Existential methods, Grief work and Working on resources* had a small effect size when rounded (*η* ^2^ = 0.009–*η* ^2^ = 0.011). It was most often applied for Initial disease (metastasized) and least for Follow‐up care. Since an initial disease that has already metastasized can be considered the most life‐threating tumor situation, interventions like *Existential methods* and G*rief work* that include talking about death and coping with loss [12] seem adequate, whilst these interventions do not apply to patients in follow‐up care who most likely have already survived cancer.

### 4.1 Implications

Our study depicts the overall need for psycho‐oncological interventions. Although there are some interventions that are more often applied than others, there are interventions that are specifically applied according to needs. Based on the most common interventions, POS teams should be trained in relationship work, resource activation, and normalization in particular. Implications for clinical practice could entail special trainings according to the patient's needs. Our results could also be the basis for an optimized preparation for further psycho‐oncological contacts in order to improve psycho‐oncological care. However, further research is necessary to generalize these results to other hospitals as well as other settings.

### 4.2 Limitations

Some limitations to our study need to be mentioned. The present study is a retrospective, cross‐sectional, and exploratory study, which limits its generalizability. Furthermore, given its exploratory nature, multiple comparisons were made without correcting for these multiple comparisons. This increases the likelihood of false positive results. The assessment of applied interventions was only restricted to one single acute care institution, so generalizability is limited. Here, the POS is established both as a liaison and consultation service. The manner of how the POS is established can affect the duration and frequency of contacts [14] and therefore the type and number of interventions used.

Other limitations concern for example missing context variables that influence the type of applied interventions that were not considered in our study, such as personal preference and experience of the therapist or the emotional, physical and/or cognitive capacity of the patient for certain interventions as well as the severity of the illness‐related somatic and psychosocial burden. Finally, the frequency and type of applied interventions in our study does not allow any conclusions regarding the efficacy of psycho‐oncological support, since there is a wide array of factors that might play a role.

## 5 Conclusion

In summary, there are interventions that are inherent to nearly all psycho‐oncological contacts, such as *Establishing a relationship* and *Exploring the current state,* creating a basis and framework for psycho‐oncological support. *Working on resources* is another intervention especially relevant for cancer patients. All other interventions are flexibly applied in their frequency, depending on needs and characteristics of the patients. Our data show that psycho‐oncology in acute settings consists of a few universal core interventions supplemented by needs‐based methods. Standardized, differentiated training is essential for this.

## Ethics Statement

I confirm that in the course of the development of the taxonomy used for the present study, institutional ethics committee approval was obtained from the Ethics Committee of the Medical Faculty of the Friedrich‐Alexander University Erlangen‐Nürnberg (FAU).

## Consent

No written informed consent of the patients for our study was required because our study analyzed anonymously standard care data from the university hospital, patients were not explicitly asked to participate in the study.

## Conflicts of Interest

The authors declare no conflicts of interest.

## Supporting information


**Table S1:** Differences in applied interventions according to gender and correlations with age.

## References

[1] A. Mehnert , T. Hartung , M. Friedrich , et al., “One in Two Cancer Patients is Significantly Distressed: Prevalence and Indicators of Distress,” Psycho‐Oncology 27, no. 1 (2018): 75–82, 10.1002/pon.4464. 28568377

[2] A. Mehnert , E. Brähler , H. Faller , et al., “Four‐week Prevalence of Mental Disorders in Patients With Cancer Across Major Tumor Entities,” Journal of Clinical Oncology 32, no. 31 (2014): 3540–3546, 10.1200/jco.2014.56.0086. 25287821

[3] Deutsche Krebsgesellschaft , Deutsche Krebshilfe , and AWMF , Psychoonkologische Diagnostik, Beratung und Behandlung von erwachsenen Krebspatient*innen (Leitlinie Kurzversion). (2023).

[4] H. Faller , J. Weis , U. Koch , et al., “Utilization of Professional Psychological Care in a Large German Sample of Cancer Patients,” Psycho‐Oncology 26, no. 4 (2018): 537–543, 10.1002/pon.4197. 27327213

[5] S. Singer , S. Dieng , and S. Wesselmann , “Psycho‐Oncological Care in Certified Cancer Centres ‐ A Nationwide Analysis in Germany,” Psycho‐Oncology 22, no. 6 (2013): 1435–1437, 10.1002/pon.3145. 22855347

[6] A. Mehnert and U. Koch , eds., Handbuch Psychoonkologie (Hogrefe, 2016).

[7] A. Mehnert‐Theuerkauf and F. Springer , “Psychoonkologie – psychosoziale Belastungen und Versorgungsbedarfe,” Urologie 63 (2024): 878–885, 10.1007/s00120-024-02395-3. 38995422

[8] H. Faller , M. Schuler , M. Richard , U. Heckl , J. Weis , and R. Küffner , “Effects of Psycho‐Oncologic Interventions on Emotional Distress and Quality of Life in Adult Patients With Cancer: Systematic Review and Meta‐Analysis,” Journal of Clinical Oncology 31, no. 6 (2013): 782–793, 10.1200/jco.2011.40.8922. 23319686

[9] L. Cillessen , M. Johannsen , A. E. Speckens , and R. Zachariae , “Mindfulness‐Based Interventions for Psychological and Physical Health Outcomes in Cancer Patients and Survivors: A Systematic Review and Meta‐Analysis of Randomized Controlled Trials,” Psycho‐Oncology 28, no. 12 (2019): 2257–2269, 10.1002/pon.5214. 31464026 PMC6916350

[10] W. W. Tao , P. Jiang , Y. Liu , Y. Aungsuroch , and X. Tao , “Psycho‐Oncologic Interventions to Reduce Distress in Cancer Patients: A Meta‐Analysis of Controlled Clinical Studies Published in People's Republic of China,” Psycho‐Oncology 24, no. 3 (2015): 269–278, 10.1002/pon.3634. 25060151

[11] S. Michie , C. E. Wood , M. Johnston , C. Abraham , J. J. Francis , and W. Hardeman , “Behaviour Change Techniques: The Development and Evaluation of a Taxonomic Method for Reporting and Describing Behaviour Change Interventions (A Suite of Five Studies Involving Consensus Methods, Randomised Controlled Trials and Analysis of Qualitative Data),” Health Technology Assessment 19, no. 99 (2015): 1–188, 10.3310/hta19990. PMC4781650 26616119

[12] M. Madl , M. Lieb , K. Schieber , T. Hepp , and Y. Erim , “A Taxonomy for Psycho‐Oncological Intervention Techniques in an Acute Care Hospital in Germany,” Oncology Research and Treatment 44, no. 7–8 (2021): 382–389, 10.1159/000517532. 34237721

[13] J. Cohen , Statistical Power Analysis for the Behavioral Sciences, 2nd ed. (Lawrence Erlbaum Associates, 1988).

[14] M. Madl , M. Lieb , K. Schieber , and Y. Erim , “The Influence of Patient‐Related Factors on the Frequency and Duration of Psycho‐Oncological Sessions in a University Cancer Center,” Journal of Psychosocial Oncology 40, no. 3 (2022): 380–396, 10.1080/07347332.2021.1964013. 34860144

[15] B. Garssen , M. Van der Lee , A. Van der Poll , A. V. Ranchor , R. Sanderman , and M. J. Schroevers , “Characteristics of Patients in Routine Psycho‐Oncological Care, and Changes in Outcome Variables During and After Their Treatment,” Psychology and Health 31, no. 10 (2016): 1237–1254, 10.1080/08870446.2016.1204447. 27351933

[16] B. E. Wampold and C. Flückiger , “The Alliance in Mental Health Care: Conceptualization, Evidence and Clinical Applications,” World Psychiatry 22, no. 1 (2023): 25–41, 10.1002/wps.21035. 36640398 PMC9840508

[17] C. Flückiger , A. C. Del Re , B. E. Wampold , and A. O. Horvath , “The Alliance in Adult Psychotherapy: A Meta‐Analytic Synthesis,” Psychotherapy 55, no. 4 (2018): 316–340, 10.1037/pst0000172. 29792475

[18] I. Ruiz‐Rodriguez , I. Hombrados‐Mendieta , A. Melguizo‐Garín , and M. J. Martos‐Méndez , “The Importance of Social Support, Optimism and Resilience on the Quality of Life of Cancer Patients,” Frontiers in Psychology 13 (2022): 833176, 10.3389/fpsyg.2022.833176. 35356348 PMC8959607

[19] R. D. Nipp , A. El‐Jawahri , J. N. Fishbein , et al., “The Relationship Between Coping Strategies, Quality of Life, and Mood in Patients With Incurable Cancer,” Cancer 122, no. 13 (2016): 2110–2116, 10.1002/cncr.30025. 27089045 PMC5160928

[20] C. T. Cheng , S. M. Y. Ho , W. K. Liu , et al., “Cancer‐coping Profile Predicts Long‐Term Psychological Functions and Quality of Life in Cancer Survivors,” Supportive Care in Cancer 27, no. 3 (2019): 933–941, 10.1007/s00520-018-4382-z. 30088138

[21] J. Zhou , Z. Wang , X. Chen , and Q. Li , “Gender Differences in Psychosocial Outcomes and Coping Strategies of Patients With Colorectal Cancer: A Systematic Review,” Healthcare 11, no. 18 (2023): 2591, 10.3390/healthcare11182591. 37761788 PMC10530630

