# Supplementary material for: Which Psycho‐Oncological Interventions Are Applied in an Acute Care Hospital in Germany?—An Exploratory Retrospective Cross‐Sectional Single Center Study
Source: Psychooncology. 2025 Oct 1;34(10):e70284. doi: 10.1002/pon.70284 (PMC12485297; doi:10.1002/pon.70284)
Supplement: Supplementary file 1 — Table S1: Differences in applied interventions according to gender and correlations with age. [file PON-34-e70284-s001.docx]

**Supplement 1** Differences in applied interventions according to gender and correlations with age

| Intervention | Gender | |  | Age |
| --- | --- | --- | --- | --- |
|  | Male | Female |  |  |
| Introduction  Counselling | 1.15 (0.91)  0.32 (0.99) | 1.19 (0.79)  0.28 (.084) | t(2634)=-0.95, p=0.34, d=-0.038  t(2634)=1.31, p=0.18, d=0.052 | r=-0.055, **p=0.005**  r=-0.123, **p<0.001** |
| Exploration of current state  Survey of disease progression/ medical  history  Defining the therapeutic mandate  Assessment of suicidality | 1.41 (1.95)  0.91 (1.31)  1.24 (1.34)  0.04 (0.23) | 1.70 (2.18)  0.88 (1.32)  1.47 (1.59)  0.03 (0.21) | t(2410)=-3.45, **p<0.001**, d=-0.134  t(2634)=0.623, p=0.533, d=0.025  t(2481)=-3.92, **p<0.001**, d=-0.151  t(2634)=0.797, p=0.426, d=0.032 | r=-0.128, **p<0.001**  r=-0.085, **p<0.001**  r=-0.079, **p<0.001**  r=-0.030, p=0.129 |
| Psycho-education  Working on emotions  Using cognitive methods  Relaxation techniques/ Guided imagery  Working on resources  Development of social skills | 0.33 (0.97)  0.19 (0.77)  0.29 (1.24)  0.03 (0.21)  0.78 (1.41)  0.12 (0.50) | 0.30 (0.76)  0.14 (0.66)  0.33 (0.93)  0.07 (0.37)  1.00 (1.67)  0.10 (0.55) | t(2634)=0.752, p=0.452, d=0.030  t(1996)=1.56, p=0.119, d=0.064  t(2634)=-1.12, p=0.263, d=-0.045  t(2594)=-3.08, **p<0.001**, d=-0.123  t(2634)=-3.59, **p<0.001**, d=-0.143  t(2634)=0.899, p=0.369, d=0.036 | r=-0.148, **p<0.001**  r=-0.059, **p=0.002**  r=-0.103, **p<0.001**  r=-0.108, **p<0.001**  r=-0.115, **p<0.001**  r=-0.035, p=0.073 |
| Accompaniment  Crisis intervention  Life review  Bearing of emotions (Holding/ Containing)  Coping with fear of progression/ recurrence  Existential methods  Grief work | 0.05 (0.30)  0.009 (0.10)  0.06 (0.25)  0.56 (1.30)  0.09 (0.45)  0.05 (0.31)  0.12 (0.58) | 0.05 (0.34)  0.013 (0.13)  0.04 (0.26)  0.57 (1.22)  0.15 (0.67)  0.05 (0.44)  0.10 (0.56) | t(2634)=-0.149, p=0.881, d=-0.006  t(2634)=-0.796, p=0.426, d=-0.032  t(2283)=1.456, p=0.146, d=0.058  t(2634)=-0.313, p=0.754, d=-0.012  t(2633)=-2.59, **p=0.009**, d=-0.096  t(2634)=0.282, p=0.778, d=0.011  t(2634)=1.04, p=0.299, d=0.041 | r=-0.055, **p=0.005**  r=-0.074, **p<0.001**  r=0.053, **p=0.006**  r=-0.095, **p<0.001**  r=-0.069, **p<0.001**  r=-0.043, **p=0.026**  r=-0.069, **p<0.001** |
| Establishing the relationship  Normalizing  Validating | 1.38 (1.89)  1.02 (1.65)  1.30 (1.89) | 1.67 (2.10)  1.35 (1.87)  1.58 (2.12) | t(2401)=-3.73, **p<0.001**, d=-0.145  t(2427)=-4.89, **p<0.001**, d=-0.190  t(2414)=-3.63, **p<0.001**, d=-0.141 | r=-0.131, **p<0.001**  r=-0.118, **p<0.001**  r=-0.113, **p<0.001** |

Note: All significant results (p<.05) are depicted in bolt.
